# Liquid biopsy and glioblastoma

**DOI:** 10.37349/etat.2023.00121

**Published:** 2023-02-25

**Authors:** Robert H. Eibl, Markus Schneemann

**Affiliations:** 1 c/o M. Schneemann, Department of Internal Medicine, Hospitals of Schaffhausen, 8208 Schaffhausen, Switzerland; 2 Department of Internal Medicine, Hospitals of Schaffhausen, 8208 Schaffhausen, Switzerland; Tianjin Medical University General Hospital, China

**Keywords:** Liquid biopsy, glioblastoma, cell-free DNA, circulating tumor DNA, minimal residual disease, monitoring, circulating tumor cells, treatment response

## Abstract

Glioblastoma is the most common and malignant primary brain tumor. Despite a century of research efforts, the survival of patients has not significantly improved. Currently, diagnosis is based on neuroimaging techniques followed by histopathological and molecular analysis of resected or biopsied tissue. A recent paradigm shift in diagnostics ranks the molecular analysis of tissue samples as the new gold standard over classical histopathology, thus correlating better with the biological behavior of glioblastoma and clinical prediction, especially when a tumor lacks the typical hallmarks for glioblastoma. Liquid biopsy aims to detect and quantify tumor-derived content, such as nucleic acids (DNA/RNA), circulating tumor cells (CTCs), or extracellular vesicles (EVs) in biofluids, mainly blood, cerebrospinal fluid (CSF), or urine. Liquid biopsy has the potential to overcome the limitations of both neuroimaging and tissue-based methods to identify early recurrence and to differentiate tumor progression from pseudoprogression, without the risks of repeated surgical biopsies. This review highlights the origins and time-frame of liquid biopsy in glioblastoma and points to recent developments, limitations, and challenges of adding liquid biopsy to support the clinical management of glioblastoma patients.

## Introduction

Glioblastoma is the most common malignant brain tumor with most patients dying within 1 year after diagnosis [1, 2]. More than a century ago Cushing introduced modern neurosurgery and developed with Bailey [3] a classification of brain tumors by coining the term “glioblastoma multiforme” (GBM). Despite optimized radio- and chemotherapy, much further progress seems to be challenging when it comes to the efficient treatment of glioblastoma patients and overall survival (OS) (Table 1). Recently, neuropathologists pioneered a revolutionary paradigm shift to improve brain tumor diagnostics [4, 5]. For over a century, glioblastomas were classified by their histological hallmarks, including necrosis and/or proliferation of the microvasculature as well as rapid infiltration of surrounding tissue. Primary glioblastomas were considered to appear *de novo*, i.e. with no detectable precursor tumor, whereas secondary glioblastomas were considered originating from a low-grade astrocytoma (II) or an anaplastic astrocytoma (III) [6]. Primary and secondary glioblastomas appeared to bear mutually exclusive gene alterations, like epidermal growth factor receptor (EGFR) overexpression and tumor protein P53 (TP53) mutations, reflecting two distinct tumor entities with different biological behavior and clinical prognosis [7].

**Table 1.** Developments related to glioblastoma diagnostics and liquid biopsy

**Year**	**Author**	**Probe**	**Method**	**Tumor**	**Milestone**
1926	Bailey and Cushing [3]	Tumor resection	Neurosurgery, histology, classification	GBM	Developed modern neurosurgery and classification of brain tumors, coined the term “glioblastoma multiforme”
1991, 1992	Eibl and Wiestler [8], Eibl et al. [9], Wiestler et al. [10]	Animal models	Oncogene transfer into neural grafts	Gliomas, PNETs	Rat tumor models, (reviewed in [11])
1992	von Deimling, Eibl et al. [12]	Frozen tumor sample	SSCP	Astrocytoma II, III	TP53 mutations are not a late event in astrocytic tumor development
1993	Louis et al. [13]	Frozen tumor sample	SSCP	Astrocytoma II, III GBM	TP53 mutations in astrocytic tumors, incl. GBM
1993	Ohgaki et al. [14]	Frozen tumor sample	SSCP	PA I	Absence of TP53 mutations in pilocytic astrocytoma
2003	Balaña et al. [15]	ctDNA	PCR to detect methylated MGMT	GBM	Methylated MGMT predicts response to alkylating chemotherapy
2014	Bettegowda et al. [16]	ctDNA	Digital PCR, sequencing	Different cancers, incl. glial tumors	ctDNA detection
2014	Sullivan et al. [17]	CTC	Removing leukocytes from blood	GBM	Detection of CTCs in GBM
2016	Louis et al. [4]	Tumor sample	Transcriptome	Nervous system tumors	Paradigm shift in diagnostics from histology to transcriptomics
2016	Underhill et al. [18]	ctDNA	Experimental study	Human GBM cells in rat brain	Fragmentomics: ctDNA fragments are shorter (134–144 bp) than normal cfDNA (167 bp)
2016	Donaldson and Park [19]	ctDNA	Observational study	NSCLC	FDA approval [20] for mutated EGFR test on liquid biopsy
2017	Yasui et al. [21]	EV	Nanowire	GBM	Detection of EVs in urinary
2021	Louis et al. [5]	Tumor sample	Transcriptome, methylome	NEW: astrocytoma IV (formerly secondary glioblastoma) and GBM	WHO classification: introducing astrocytoma IV and molecular definition of GBM (even without typical histological features)

CTC: circulating tumor cell; EV: extracellular vesicle; PNETs: primitive neuroectodermal tumors; SSCP: single-strand conformation polymorphism; PA I: pleomorphic adenoma I; PCR: polymerase chain reaction; ctDNA: circulating tumor DNA; MGMT: O^6^-methylguanine-methyltransferase; cfDNA: cell-free DNA; NSCLC: non-small cell lung cancer; FDA: Food and Drug administration; WHO: World Health Organization

Further molecular characterization led to the paradigm shift in diagnostics: typical hallmarks of glioblastoma, like endothelial proliferation or necrosis, are not necessary to consider a tumor formerly diagnosed as astrocytoma WHO grade II or III as a molecularly defined glioblastoma. Therefore, the current WHO classification from 2021 restricts the term glioblastoma only to the former group of primary glioblastomas, also including a few low-grade and anaplastic astrocytomas with a corresponding mutational status [5]. This rather revolutionary, new classification system is intended to dissect different oncogenic pathways of biologically distinct tumor entities to improve clinical decision-making, although the current therapeutic options of chemo- and radiotherapy remain poor. This may change with a better understanding of the tumor entities, earlier diagnosis, and monitoring of treatment and resistance as well as the development of new therapeutic approaches, including immune targeting therapies [22].

**Figure 1.**
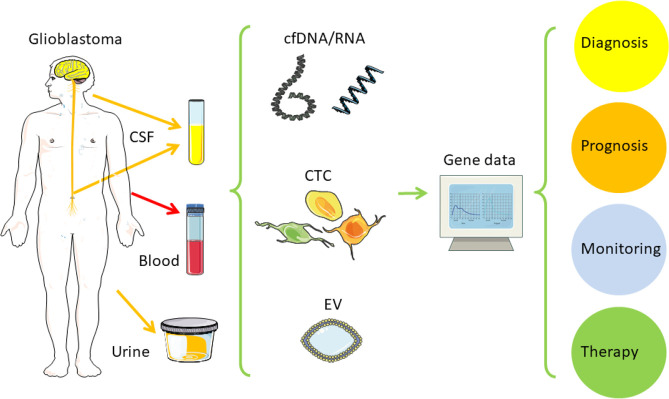
Liquid biopsy in glioblastoma. This figure contains modified images from Servier Medical Art (https://smart.servier.com) licensed by a Creative Commons Attribution 3.0 Unported License *Note.* Adapted from “Liquid biopsy and primary brain tumors,” by Eibl RH, Schneemann M. Cancers (Basel). 2021;13:5429 (https://doi.org/10.3390/cancers13215429). CC BY; “Liquid biopsy for monitoring medulloblastoma,” by Eibl RH, Schneemann M. Extracell Vesicles Circ Nucl Acids. 2022;3:263–74 (http://dx.doi.org/10.20517/evcna.2022.36). CC BY.

For glioblastoma and other brain tumors, ctDNA harvested from cerebrospinal fluid (CSF) leads to higher sensitivity than blood or urine (Table 2) [23–27]. In this regard, CSF-ctDNA also represents the genomic mutations better and is the method of choice to use higher sensitivity to detect actionable mutations and copy number aberrations [CNA; EGFR, phosphatase and tensin homolog (PTEN), estrogen receptor 1 (ESR1), isocitrate dehydrogenase 1 (IDH1), erb-b2 receptor tyrosine kinase 2 (ERBB2), fibroblast growth factor receptor 2 (FGFR2), MGMT] [28–31]. These tools of precision oncology support better prognosis, clinical decision-making, treatment as well as monitoring, and new immune therapies [22]. Combining the new molecular classification from solid tumor samples with the potential of liquid biopsy should allow better monitoring of glioblastoma development and treatment response. It helps to avoid fine-needle aspiration (FNA) cytology and stereotactic surgical biopsies, thus reducing the risk of infection and brain damage [32]. Distant from the original tumor mass, cell-free nucleic acids (cfDNA/RNA), EV, or CTC can be found in body fluids, such as blood, CSF, or even urine (Figure 1). Liquid biopsy is currently used in observational and interventional studies with glioblastoma patients for monitoring tumor development or treatment response. This will help to improve clinical decisions. Here the authors summarize the application potential of liquid biopsy in glioblastoma and what will be needed to include this in clinical routine.

**Table 2.** Origins of testing ctDNA markers in glioblastomas

**Year**	**Gene**	**Variation**	**Source**	**Method**	**Tumor**
2003 [15]	*MGMT*	Methylation	Serum	MS-PCR	GBM
2006 [33]			Plasma	MS-PCR	GBM, AA
2010 [34]			Serum	MS-PCR	Astrocytic tumors (WHO III, IV), oligodendroglial tumors (WHO II, III)
2013 [29]			Serum	MS-PCR	Glial tumors (II, III, IV), meningioma
2003 [15]	*p16*	Methylation	Serum	MS-PCR	GBM
2006 [33]			Plasma	MS-PCR	GBM, AA, AOA
2003 [15]	*DAPK*	Methylation	Serum	MS-PCR	GBM
2003 [15]	*RASSF1A*	Methylation	Serum	MS-PCR	GBM
2013 [29]					Glial tumors (II, III, IV), meningioma
2006 [33]	*p73*	Methylation	Plasma	MS-PCR	GBM
2010 [34]	*PTEN*	Methylation	Serum	MS-PCR	Astrocytic tumors (WHO III, IV)
2014 [17]		Mutation	Plasma, serum	Digital PCR, sequencing	Glioma II, AA, GBM
2010 [34]	*10q*	LOH	Serum	LOH	Astrocytic (WHO III, IV), oligodendroglial (WHO II, III)
2012 [35]	*IDH1*	Mutation (R132H)	Plasma	Digital PCR	Glioma (WHO grade II, III, IV)
2014 [16]		Mutation	Plasma, serum	Digital PCR, sequencing	Glioma II, AA, GBM
2013 [29]	*p15INK4B*	Methylation	Serum	MS-PCR	Glial tumors (II, III, IV), meningioma
2013 [29]	*p14ARF*	Methylation	Serum	MS-PCR	Glial tumors (II, III, IV), meningioma
2014 [16]	*TP53*	Mutation	Plasma, serum	Digital PCR, sequencing	Glioma II, AA, GBM
2014 [16]	*EGFR*	Mutation	Plasma, serum	Digital PCR, sequencing	Glioma II, AA, GBM
2014 [16]	*PIK3CA*	Mutation	Plasma, serum	Digital PCR, sequencing	Glioma II, AA, GBM
2015 [28]	*TP53, EPHB1, TERT, PIK3CG, IDH1, ANK, EGFR, PTEN, FTH1, OR51D1*	Mutation	CSF (plasma)	ddPCR, MAF	GBM
2015 [27]	Genome	Mutation	CSF	TAS/WES	AA III, PA I, ependymoma, medulloblastoma IV, GBM, LGG II, diffuse astrocytoma
2017 [36]	Gene panels (54, 68, 70 genes) including *TP53*, *EGFR*, *MET*	Mutation	Plasma	NGS	Brain tumors (not specified)
2018 [23]	*IDH1*, *IDH2*, *TP53*, *TERT*, *ATRX*, *H3F3A*, *HIST1H3B*	Mutation	CSF	Sequencing	Diffuse gliomas
2018 [25]	Genome	SCNAs and fragmentation	CSF	WGS	Glioma
2018 [37]	*TERT*	Mutation	CSF (plasma)	PCR, sequencing	GBM
2019 [38]	Genome including *TP53*, *JAK2*, *NF1*, *EGFR*, *BRAF*, *IDH1, NRAS*, *GNAS*, *ATM*	Mutation	Plasma	NGS	Astrocytic/oligodendral tumors grades I–IV, including GBM, medulloblastoma, meningioma, and ependymoma
2019 [24]	P19Q, *IDH1*, *CIC*, *ATRX*, *TP53*	Mutation	CSF	NGS	LGG, GBM

MS-PCR: methylation-sensitive PCR; AA: anaplastic astrocytoma; AOA: anaplastic oligoastrocytoma; *DAPK*: death-associated protein kinase; *RASSF1A*: ras association domain family 1 isoform A; LOH: loss of heterozygosity; *PIK3CA*: phosphatidylinositol-4, 5-bisphosphate 3-kinase catalytic subunit alpha; *PIK3CG*: phosphatidylinositol-4, 5-bisphosphate 3-kinase catalytic subunit gamma; *EPHB1*: ephrin type-b receptor 1; *OR51D1*: olfactory receptor family 51 subfamily D member 1; *TERT*: telomerase reverse transcriptase; *ANK*: amplified natural killer; *FTH1*: ferritin heavy chain 1; ddPCR: droplet digital PCR; MAF: mutant allelic frequency; TAS: targeted amplicon sequencing; WES: whole exome sequencing; LGG: low-grade glioma; NGS: next generation sequencing; *ATRX*: alpha-thalassemia/mental retardation syndrome X-linked; *H3F3A*: histone 3.3 gene variant A; *HIST1H3B*: histone 3.1 gene; SCNA: somatic copy-number alterations; WGS: whole-genome sequencing; *JAK2*: janus kinase 2; *NF1*: neurofibromatosis type 1; *BRAF*: v-Raf murine sarcoma viral oncogene homolog B; *NRAS*: neuroblastoma RAS (viral oncogene homolog of rat sarcoma virus); *GNAS*: G protein alpha(s); *ATM*: Ataxia telangiectasia mutated; 1P19Q: 1p/19q codeletion of chromosome arms; CIC: capicua (Catalan: head-and-tail), a Drosophila homologue gene for a transcription repression factor

## Liquid biopsy

Within the last 20 years, various methods were developed and applied which can be summarized with the term “liquid biopsy” (Figure 1) [39–46]. Basically, tumor cells or tumor derived nucleic acids, proteins, or EV can be detected in bodily fluids, which are repeatedly accessible and at a lower risk compared to tissue biopsy. For most cancers, blood derived serum or plasma serves as the main source, although CSF, when available, appears to be the better option for brain tumors. The blood brain barrier (BBB) is assumed deterring tumor cells from entering the bloodstream. CSF offers another advantage of less background from leukocytes or cfDNA. An overview on glioblastoma research leading to the current diagnostic classification and liquid biopsy is shown in Table 1.

**Figure 2.**
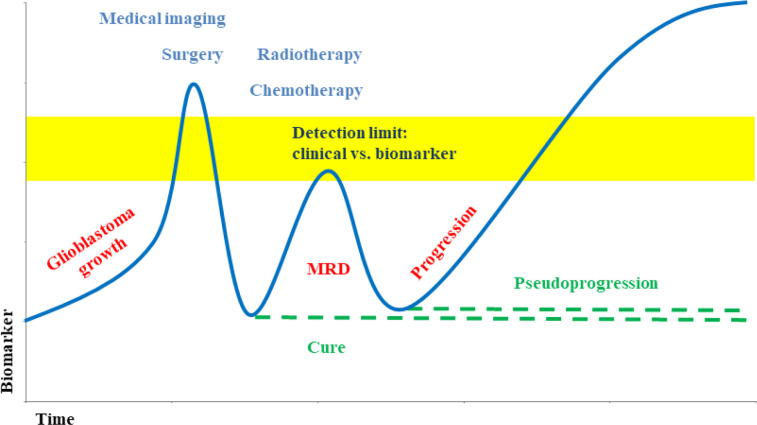
Biomarker level during glioblastoma development and therapy [40, 46]. Liquid biopsy supports earlier detection of minimal residual disease (MRD) and allows differentiating between progression and pseudoprogression *Note.* Adapted from “Liquid biopsy and primary brain tumors,” by Eibl RH, Schneemann M. Cancers (Basel). 2021;13:5429 (https://doi.org/10.3390/cancers13215429). CC BY; “Liquid biopsy for monitoring medulloblastoma,” by Eibl RH, Schneemann M. Extracell Vesicles Circ Nucl Acids. 2022;3:263–74 (http://dx.doi.org/10.20517/evcna.2022.36). CC BY.

The tumor derived ctDNA can typically be found in a range from 1% to 10% of the total cfDNA. Tumor growth and metastatic spread often lead to higher levels of the biomarker (Figure 2, Table 2) [40], whereas removal of the tumor, as well as treatments with irradiation and chemotherapy typically reduce the ctDNA. However, a lack of ctDNA decrease points to a lack of treatment response and an already resistant tumor. Resistance development by clonal selection of resistant tumor cells can be monitored by an initial decrease with therapy, but a later increase of ctDNA.

The average size of (tumor-derived) cfDNA fragments from blood is slightly shorter than the size of normal background cfDNA. Underhill and colleagues [18] showed this in a xenograft model implanting human-derived glioblastoma stem-cell like cell lines into the nude rat brain, which led to shorter principal fragment sizes of 134–144 bp of the tumor-derived cfDNA compared to 167 bp of the background (normal) cfDNA [18]. Currently, transcriptomics for the detection of sequence mutations, and methylomics for the epigenetic signature of tumors lead to clinically most promising approaches in liquid biopsy of glioblastoma.

With CellSearch in 2004, the detection of CTCs was approved for clinical use as an independent and predictive marker of carcinomas, incl. prostate, breast, ovarian, colorectal, lung, and other cancers. Unfortunately, glioblastoma and other brain tumor cells don’t share the epithelial marker used in this detection system. In 2014, Sullivan and colleagues [17] were able to detect rare CTCs at a surprisingly high frequency of 13 of 33 (39%) glioblastoma patients [17, 47–52]. This finding was surprising since hematogenous metastasis is described as extremely uncommon for glioblastoma. The authors used a microfluidic device and a negative selection strategy to remove leukocytes from blood. The short OS of glioblastoma patients often less than one year may not allow micro-metastases to grow to larger metastases, although the accelerated growth leads to a high frequency of tumor cells entering the blood stream.

Brain tumors were considered to metastasize via the CSF to other regions of the brain and the spinal cord. This well-established assumption was recently challenged by CTCs from medulloblastoma patients [53], which were able to spread via the blood in a parabiotic xenograft model of mice to form leptomeningeal metastases. A chemokine and its receptor were identified to drive this leptomeningeal homing. Eibl postulated already in 2000 [54] a similar mechanism for organ-specific metastasis, thus metastatic tumor cells may share similar adhesion steps and receptors, including chemokines, with homing lymphocytes. With colleagues from biophysics Eibl further investigated this theory in the following years [55–67]. Several of these tumor cell rolling and arrest models were found and further analyzed at the so-called single-molecule level on living cells with atomic force microscopy (AFM), but not yet with medulloblastoma or glioblastoma cells.

EVs as cell-derived, small vesicles that contain nucleic acids and proteins, which can serve as potential biomarkers. In contrast to blood with many leukocytes CSF offers a better signal-to-noise ratio [68]. A nanowire scaffold allowed the detection of EVs from the urine of glioblastoma patients [21].

MicroRNAs (miRNAs) are small, non-coding RNA molecules with 20–24 bp of length. They can regulate and stabilize messenger RNA (mRNA). miRNAs are involved in tumor biology, angiogenesis, and immunology. In glioblastomas, miRNAs are considered biomarkers, but also therapeutic targets [69]. miRNAs were also detected in urine to confirm different central nervous system (CNS) tumors, including glioblastoma [70].

An increasing number of research institutions share their data to allow data-mining and meta-analysis. For comparing such data they should meet the findable, accessible, interoperable, and reusable (FAIR) principles of the data [71].

## Clinical studies

Only a limited number of ongoing studies, mainly from Canada, China, France, Switzerland, the UK, and the USA, evaluate the clinical use of liquid biopsy for glioblastoma patients (Table 3). Most of the observational studies use standard and routine blood drawing as the source for ctDNA to compare the molecular profile with the molecular diagnosis from tissue biopsy. In some cases, and only when routinely available, CSF analysis can be added for comparison. Other interventional studies include liquid biopsy only as an additional tool to monitor tumor response after treatments (Table 3). In an ongoing interventional phase I trial at the University of California, Los Angeles (UCLA) Jonsson Comprehensive Cancer Center, California, USA patients with recurrent glioblastoma are monitored by liquid biopsy of a treatment, which combines a monoclonal antibody and a vaccine (NCT04201873) [72]. Changes of gene expression signatures from the archival tumor as well as from peripheral blood before and after treatment will be associated with clinical outcomes [progression-free survival (PFS) and OS] (Table 3). With 1,000 participants with gliomas grades II–IV in the ongoing British Tessa Jowell BRAIN MATRIX study (NCT04274283) [73], the feasibility of molecular stratification and targeted therapy will be addressed to optimize the clinical management of patients with glioma by enhancing clinical outcomes and reducing avoidable toxicity, improving the management of post-operative residual and recurrent disease and improving survivorship. This includes molecular analysis by both WES and epigenomic classification of matched tissue and blood samples, but not CSF, for the detection of targetable mutations in the tumor or the germline. CircTeloDIAG is an ongoing observational study at the Lyon Civil Hospices (Hospices Civils de Lyon), Lyon, France, for 150 participants with magnetic resonance imaging (MRI) suspected or recurrent glioma grades II–IV, including glioblastomas (Table 3). The rationale is to establish liquid biopsy for routine diagnosis and monitoring of gliomas. Therefore, the study aims to detect and monitor three oncogenic markers: IDH mutation, TERT mutation, and ATRX. The investigators expect circTeloDIAG to improve and accelerate the current classification of gliomas. This combination of three biomarkers may be approved as a versatile tool for detecting and monitoring all types of gliomas with routine liquid biopsy (NCT04931732) [74]. The ongoing multi-center PLANET study from France aims for sequential analysis of tumor and liquid biopsies of 500 cancer patients, including patients with GBM, chronic lymphocytic leukemia (CLL), and advanced/metastatic solid tumors. To identify prognostic and predictive biomarkers for GBM changes on ctDNA from the blood before and after standard chemotherapy will be analyzed (NCT05099068) [75]. Although no glioblastoma from adults was included in a study from Pagès [76] investigating over 200 pediatric CNS tumors of different grades and malignancy the results support the use of liquid biopsy for the highly malignant tumors in the brain. A study from Switzerland aims to validate a new PCR-based method for cheaper and improved analysis [77]. The number of participants is not disclosed (NCT04539431). In an ongoing diagnostic study in 15 locations in the USA, the amount of cfDNA from 57 glioblastoma patients will be monitored from the blood before and after physical treatment with a microbubble resonator (Exablate Model 4000). The investigators expect at least a 2-fold increase in harvesting cfDNA at 1 h post BBB disruption (BBBD, NCT05383872) [78]. Diffuse low-grade gliomas (DLGG, or WHO grade II gliomas) are different to highly malignant glioblastomas, but may continuously progress to grade III or IV tumors. An exploratory study in Montpellier, France is pioneering a challenging approach to search for CTCs in blood from DLGG patients, but also investigates the tumor-educated blood platelets (TEP) and three biomarkers for oncogenic pathways (IDH, 1p19q, ATRX, NCT05133154) [79]. An observational study from Wuhan, China with 500 participants combines the new molecular classification of gliomas with liquid biopsy and deep-learning MRI radiomics to predict glioma grading and molecular subtyping (NCT05536024) [80]. Several other intended studies on glioblastoma and ctDNA were terminated or withdrawn by the investigators due to either the SARS-CoV-2 pandemic [81], lack of funding [82], or other undisclosed reasons [83]. They mainly aimed for correlating primary tumors with ctDNA mutations in blood [84]. Several studies searching for CTCs didn’t post any results [85, 86]. Before it became clear that, surprisingly, CTCs can be found regularly in only rarely metastatic glioblastomas, one early study addressed this question. Blood was analyzed before and shortly after the operation of 25 glioblastoma patients, but no results were posted to the clinical study (NCT00001148) [85]. Another study using liquid biopsy of glioblastomas involved 130 glioblastoma patients investigating the antigen profile of CTCs with a cluster of differentiation (CD) signature [86].

**Table 3.** Current clinical studies using liquid biopsy in glioblastomas

**Year**	**Study**	**Tumor**	**Name/Method**	**Outcome measures**
2020–2024	NCT04201873 [72] Interventional phase I	40 Recurrent GBM	RNA-seq and nanoString IO360	Other outcome/measures: gene expression signature from peripheral blood before/after treatment
2020–2025	NCT04274283 [73] Observational	1,000 Glioma II–IV	Tessa Jowell BRAIN MATRIX, whole genome and epigenomic classification	Matching tumor and blood, but not CSF, for targetable mutations
2021–2024	NCT04931732 [74] CircTeloDIAG Observational	150 MRI suspected: glioma II–IV, incl. GBM	CicTeloDIAG 3 oncogenic pathways: *IDH* *TERT* *ATRX*	Validate liquid biopsy for glioma II–IV; blood ctDNA (also from CTCs) for diagnosis and monitoring of relapse
2021–2025	NCT05099068 [75] Interventional	500 Advanced/metastatic tumors, GBM, CLL	PLANET sequential liquid biopsy, WES, RNA-seq	Monitoring changes in the genetic profile of GBM after chemotherapy
2022	Pagès et al. [76]	258 CNS tumors, incl. HGG (no adult GMB)	ULP-WGS	ctDNA detection: CSF > blood, not in urine. Liquid biopsy is useful for high-grade tumors
2022	NCT04539431 [77] Observational	220 Glioma	SensiScreen glioma	Validation of cheaper, more sensitive PCR-platform for liquid biopsy (blood and CSF), comparison with tissue
2022–2023	NCT05383872 [78] Diagnostic	57 GBM	Microbubble resonator (Exablate Model 4000)	BBBD or liquid biopsy expected to increase cfDNA
2022–2024	NCT05281731 [87] Interventional	20 GBM	Sonobiopsy device testing, blood ctDNA, deep sequencing	Matching mutations post-sonobiopsy and tumor tissue
2022	NCT05133154 [79] Exploratory	50 Participants, DLGG (30 low-grade, 10 high-grade glioma, 10 controls)	Liquid biopsy in low-grade glioma, CTC, TEP	Search for CTCs (> 0), TEP in blood, evaluation of blood-based biomarkers (IDH, 1p19q, ATRX) for diagnosis and monitoring
2022–2023	NCT05536024 [80] Observational	500 Glioma	Liquid biopsy, deep learning MRI	Prediction of glioma grading and molecular subtype

IO360: Immuno-Oncology 360°; HGG: high-grade glioma; ULP-WGS: ultra-low-pass whole-genome sequencing

## Conclusions

For over a century treatment options, as well as the OS remain limited for glioblastoma patients. A recent paradigm shift in diagnosing glioblastomas by their genetic profile, and the newly established entity of astrocytoma IV—can be combined with different methods of liquid biopsy from CSF or blood, and to a lesser extent also from urine. Gene alterations detected in ctDNA mirror the heterogeneity of the original tumor and allow an accurate molecular diagnosis with follow-ups to monitor tumor and resistance development. ctDNA can detect MRD earlier which may open a time-frame, especially for emerging new potential therapeutic options with immune or vaccination therapies. Low amounts of ctDNA can challenge sensitivity and may be overcome with further development steps in technology. Currently, a rising number of clinical studies in several countries use ctDNA mainly from blood to match the molecular profile with the tissue biopsy. Other clinical studies include liquid biopsy from ctDNA as monitoring of treatment response, i.e. it is already accepted that such an approach is reasonable for glioblastomas and other brain tumors [40, 88]. CTCs were surprisingly detectable in glioblastomas, and at a high frequency, but this appears to be a major challenge and will probably be restricted to only a few highly specialized research centers to improve the methodology and develop versatile standards. Since glioblastoma cells are derived from neural tissue they lack the epithelial marker used otherwise for the enrichment of epithelial-derived carcinoma cells. Adding another selection or enrichment marker to the CellSearch system may help. This may include variants of CD44 [52]. The full diagnostic and prognostic potential of CTCs may be discovered by analysis with AFM-based pharmacology studies at the single-molecule level [65]. One recently started a clinical study on CTCs and a subset of glioma appears to be very challenging. The study aims to detect CTCs from DLGG, which are mainly low-grade tumors and quite different from highly malignant glioblastomas, but which can develop into more malignant tumors and likely will allow a longer observation time. This implies that the investigators expect to be able to detect CTCs even in low-grade gliomas, which should support such an approach for glioblastomas as well. Epigenetic markers, but also specific miRNAs may be included in future studies. Continuing technology improvement and reduction of artifacts offer new chances to further improve the sensitivity of liquid biopsy from CSF, blood, and urine. One optimistic view may include the use of liquid biopsy as a diagnostic tool to detect and target druggable mutations even prior to neurosurgical removal of the tumor, thus leading to a reduction of the tumor mass and facilitating the operation and improving OS. Some of the intended clinical studies were withdrawn or terminated prior to finishing due to the current challenges within the Covid19 pandemic and lack of funding. Altogether, several methods of liquid biopsy of glioblastoma are entering the clinic, ctDNA has been shown as a versatile biomarker for glioblastoma monitoring in both observational and interventional clinical studies, but further studies need to establish suitable protocols and validate new gold standards. With further improvement of technology, sensitivity is still expected to increase, whereas specificity appears to be already sufficient.

After a century, with milestones in neurosurgery, irradiation and chemotherapy, the recent paradigm shift in diagnostics to a new, molecular classification boosts another milestone: liquid biopsy, with ctDNA from CSF or blood, is already applied in an emerging number of clinical studies and almost ready to enter routine applications. With expected advances in technology, CTCs will also serve as promising biomarkers for early diagnosis and better disease and treatment monitoring and are likely to improve the clinical management of these devastating brain tumors. The other major challenge then still will remain: the limitation of treatment choices. New attempts, however, based on immunology to target glioblastoma are promising.

## Abbreviations

ATRX: alpha-thalassemia/mental retardation syndrome X-linked
cfDNA: cell-free DNA
ctDNA: circulating tumor DNA
CNS: central nervous system
CSF: cerebrospinal fluid
CTC: circulating tumor cell
DLGG: diffuse low-grade glioma
EGFR: epidermal growth factor receptor
EV: extracellular vesicles
GBM: glioblastoma multiforme
IDH: isocitrate dehydrogenase
MGMT: O^6^-methylguanine-methyltransferase
miRNA: microRNA
MRI: magnetic resonance imaging
MS-PCR: methylation-sensitive polymerase chain reaction
OS: overall survival
PCR: polymerase chain reaction
PTEN: phosphatase and tensin homolog
TEP: tumor-educated platelets
TERT: telomerase reverse transcriptase
TP53: tumor protein P53
WES: whole exome sequencing
WHO: World Health Organization

## Declarations

### Copyright

© The Author(s) 2023.
